# Acute Appendicitis During Pregnancy: A Seven-Year Retrospective Case Series at a United Kingdom Tertiary Hospital

**DOI:** 10.7759/cureus.119324

**Published:** 2026-11-08

**Authors:** Aishwarya R Kadrekar, Sanjay Rao, Ojas Gala

**Affiliations:** 1 Obstetrics and Gynaecology, James Cook University Hospital, Middlesbrough, GBR; 2 Obstetrics and Gynaecology, Seth Gordhandas Sunderdas Medical College, Mumbai, IND

**Keywords:** appendectomy, appendicitis, magnetic resonance imaging, postpartum period, pregnancy, retrospective study

## Abstract

Background

Acute appendicitis is the most common surgical problem encountered during pregnancy. Our case series was conducted to evaluate the clinical presentation, diagnosis, management, and outcomes of acute appendicitis in pregnant women.

Methods

A retrospective analysis of nine pregnant women diagnosed with acute appendicitis at James Cook University Hospital, United Kingdom, from 2017 to 2024 was conducted. Patients were evaluated according to age, clinical presentation, gestational age, diagnostic modalities, histopathology, time interval from admission to surgery, type of surgery, outcomes, and complications.

Results

The mean age was 26.6 years. Cases occurred in the first trimester (n = 1/9, 11.1%), second trimester (n = 3/9, 33.3%), third trimester (n = 4/9, 44.4%), and postpartum period (n = 1/9, 11.1%). Right iliac fossa pain was the most common presentation. WBC counts and CRP levels were elevated in all cases. Imaging was used to support the diagnosis in 7/9 patients (77.8%). Ultrasound was performed in 5/9 patients (55.6%), yielding positive findings in 1/5 (20.0%), inconclusive findings in 1/5 (20.0%), and false-negative findings in 3/5 (60.0%). MRI was performed in 7/9 patients (77.8%) and identified appendicitis in all clinically suspected cases, although 2/5 examinations (40.0%) were subsequently considered false-positive. The mean interval from presentation to surgery was 3.4 days. All patients (n = 9/9, 100%) underwent surgery, including laparoscopic appendicectomy in 6/9 (66.7%) and open appendicectomy in 3/9 (33.3%). Histopathological confirmation of acute appendicitis was obtained in 5/9 cases (55.6%). The negative appendicectomy rate was 4/9 (44.4%). Complications among confirmed cases included peritonitis in 5/9 patients (55.6%), sepsis in 2/9 (22.2%), perforation in 1/9 (11.1%), and preterm labour in 1/9 (11.1%).

Conclusion

Our case series demonstrates the continued challenges in the accurate and timely diagnosis of acute appendicitis, coupled with a reluctance to operate on pregnant women, which leads to delays in surgical intervention, thereby increasing the risk of complications. The surgical approach (laparoscopic/open) did not change outcomes. MRI demonstrates superior diagnostic reliability; however, despite advances in diagnostic modalities, the negative laparotomy rate remains high during pregnancy.

## Introduction

Acute appendicitis is the most common non-obstetric surgical emergency encountered during pregnancy and remains an important cause of maternal morbidity. The reported incidence ranges from approximately 1 in 500 to 1 in 2,000 pregnancies, with diagnosis often complicated by physiological and anatomical changes associated with gestation [1-3]. Delayed diagnosis and treatment may increase the risk of appendiceal perforation, peritonitis, sepsis, preterm labour, and adverse pregnancy outcomes, making timely recognition and management essential.

The diagnosis of acute appendicitis during pregnancy remains challenging because common presenting symptoms, including abdominal pain, nausea, and vomiting, frequently overlap with normal symptoms of pregnancy. Furthermore, progressive enlargement of the uterus may alter the anatomical position of the appendix and affect the reliability of physical examination findings. Although laboratory investigations such as WBC count and CRP may support clinical assessment, no individual symptom, examination finding, or inflammatory marker has demonstrated sufficient accuracy to reliably confirm or exclude appendicitis in all patients [1,4].

Imaging therefore plays a central role in the diagnostic pathway. Ultrasound is generally recommended as the initial imaging modality because it is readily available and avoids exposure to ionising radiation. However, visualisation of the appendix can be limited, particularly in advanced pregnancy, and negative or inconclusive findings do not reliably exclude disease. MRI has emerged as a valuable second-line investigation following inconclusive ultrasound findings and has demonstrated favourable diagnostic performance in published studies [5-7]. When diagnostic uncertainty persists, further imaging may be considered according to the clinical scenario and multidisciplinary assessment of maternal and fetal considerations.

Management presents an additional challenge because clinicians must balance the potential risks of operative intervention against the consequences of untreated or progressive appendicitis. Concerns regarding fetal wellbeing, diagnostic uncertainty, and the possibility of a negative appendicectomy may influence decision-making. Nevertheless, imaging findings should be interpreted in conjunction with clinical assessment, laboratory investigations, and the overall clinical trajectory of the patient rather than in isolation [2,8-10]. Timely multidisciplinary collaboration involving obstetricians, surgeons, anaesthetists, and radiologists is therefore essential in guiding investigation and treatment decisions.

Although acute appendicitis during pregnancy has been examined in previous observational studies, diagnostic uncertainty and variability in management remain important clinical challenges [11-15]. Contemporary data describing real-world decision-making, imaging performance, operative management, and patient outcomes continue to be valuable, particularly within the context of modern imaging pathways and multidisciplinary care.

The objective of this study was to evaluate the clinical presentation, diagnostic investigations, operative management, histopathological findings, and maternal outcomes of patients treated for suspected acute appendicitis during pregnancy and the postpartum period at a tertiary referral centre in the United Kingdom. Particular attention was given to the relationship between imaging findings, clinical decision-making, timing of surgical intervention, and subsequent outcomes.

## Materials and methods

Study design and setting

This case series included an analysis of the medical records of nine pregnant women diagnosed with appendicitis and managed at James Cook University Hospital, Middlesbrough, United Kingdom, from July 2017 to July 2024. The study involved collaboration between the Departments of Obstetrics and Gynaecology, General Surgery, and Anaesthesia. Pregnant women of any gestational age presenting with clinical features suggestive of appendicitis were included, while women with other causes of acute abdomen or incomplete medical records were excluded.

Data collection

Clinical information was obtained retrospectively from hospital records. The variables collected included age, gestational age or postpartum status, presenting symptoms, laboratory investigations, imaging modalities and reported findings, time from presentation to surgery, operative approach, intraoperative findings, histopathological results, complications, and maternal and neonatal outcomes.

Imaging findings were reviewed in relation to the subsequent clinical course, operative findings, and histopathological results, where available.

Outcomes

The principal outcomes were the imaging investigations undertaken and their reported findings, the interval between presentation and surgery, operative approach, histopathological confirmation of acute appendicitis, and reported complications.

Histopathological confirmation was based on the final pathology report. A negative appendicectomy was defined as removal of the appendix without histopathological evidence of acute appendicitis, subject to confirmation of the final reports.

Statistical analysis

Data were analysed descriptively. Categorical variables are presented as numbers and percentages, using the total cohort of nine patients as the denominator where data were available for all patients. Continuous variables are summarised using means and ranges, where appropriate.

## Results

Patient characteristics and diagnostic investigations are summarised in Table 1.

**Table 1:** Patient characteristics and diagnostic investigations. Data are presented as number/total (percentage), unless otherwise specified. Percentages are calculated using the total number of patients with available data for the relevant variable.

Variable	Result, n (%)
Total patients	9 (100%)
Mean age, years	26.6
First trimester	1 (11.1%)
Second trimester	3 (33.3%)
Third trimester	4 (44.4%)
Postpartum period	1 (11.1%)
Ultrasound performed	5 (55.6%)
MRI performed	7 (77.8%)
CT performed	1 (11.1%)

Nine patients were included in the series. The mean age was 26.6 years, with a range of 21-35 years. One patient presented during the first trimester, three during the second trimester, and four during the third trimester. One additional patient presented during the postpartum period.

Right iliac fossa pain was the predominant presenting symptom. Other reported symptoms included fever, nausea, and vomiting.

Inflammatory markers were elevated in all cases, with increased WBC counts and CRP levels.

Imaging investigations

Ultrasound was performed in five patients. The available case summary recorded one examination as positive, one as inconclusive, and three as false-negative in relation to the eventual diagnosis. These findings illustrate the potential limitations of ultrasound in this setting, although the small number of examinations does not permit a reliable estimate of its diagnostic accuracy. MRI showed better diagnostic accuracy, identifying appendicitis in all cases missed on ultrasound scan (USS), but was false-positive in two cases. A CT scan performed in the postnatal patient correctly identified appendicitis.

Management and operative findings

All patients were initially started on IV antibiotics; however, all eventually required surgical intervention (9/9, 100%). The time interval from presentation to surgery ranged from 1 to 8 days (mean: 3.4 days). Of these cases, 6/9 (66.7%) were performed laparoscopically, and 3/9 (33.3%) were performed using an open approach (third trimester).

Histopathology and complications

Histopathological examination confirmed acute appendicitis in 5/9 patients (55.6%), all of whom experienced complications, including localised/generalised peritonitis in 5/9 patients (55.6%), sepsis in 2/9 patients (22.2%), perforation in 1/9 patients (11.1%), or preterm labour in 1/9 patients (11.1%), highlighting the high risks and challenges associated with treating appendicitis during pregnancy.

The negative appendicectomy rate was 4/9 patients (44.4%). There were no cases of neonatal or maternal mortality.

Operative management, histopathological findings, and clinical outcomes are summarised in Table 2.

**Table 2:** Operative management and clinical outcomes. Data are presented as number (percentage), unless otherwise stated. Percentages are calculated using the total study population (n = 9), unless indicated otherwise. Clinical complications may overlap, and individual patients may have experienced more than one complication.

Variable	Result, n (%)
Surgical management	9 (100%)
Laparoscopic appendicectomy	6 (66.7%)
Open appendicectomy	3 (33.3%)
Histopathological confirmation of acute appendicitis	5 (55.6%)
Peritonitis	5 (55.6%)
Sepsis	2 (22.2%)
Appendiceal perforation	1 (11.1%)
Preterm labour	1 (11.1%)

## Discussion

This seven-year retrospective case series describes nine patients who underwent surgery for suspected acute appendicitis during pregnancy or the postpartum period at a UK tertiary hospital. Acute appendicitis during pregnancy remains a challenging clinical condition because physiological and anatomical changes associated with pregnancy may obscure classical symptoms and signs. The principal finding of this study is the degree of diagnostic uncertainty that may persist despite the use of contemporary imaging modalities. Our findings emphasise the importance of integrating clinical assessment, laboratory investigations, imaging findings, and multidisciplinary decision-making when evaluating pregnant patients with suspected appendicitis.

Limitations of imaging during pregnancy

The diagnosis of acute appendicitis during pregnancy is complicated by overlapping symptoms, physiological leukocytosis, and difficulties in visualising the appendix as gestation progresses. Ultrasound is widely accepted as the initial imaging modality because it is readily available and avoids exposure to ionising radiation. However, the appendix is frequently not visualised, and negative or inconclusive findings do not reliably exclude disease when clinical suspicion remains high. MRI is increasingly utilised as a second-line investigation following inconclusive ultrasound findings and has demonstrated favourable diagnostic performance in previous studies [5-7].

In our series, ultrasound was performed in 5/9 patients (55.6%). One examination was positive (n = 1/5, 20.0%), one was inconclusive (n = 1/5, 20.0%), and three were subsequently considered false-negative (n = 3/5, 60.0%). MRI was performed in 7/9 patients (77.8%) and identified appendicitis in all clinically suspected cases; however, 2/5 examinations (40.0%) were subsequently considered false-positive when correlated with histopathological findings. These observations highlight the limitations of relying on imaging findings in isolation. Given the small sample size, these results should be interpreted descriptively and should not be regarded as estimates of sensitivity, specificity, or comparative diagnostic accuracy.

Only one postpartum patient underwent CT imaging (n = 1/9, 11.1%), which correctly identified appendicitis. Given the limited number of CT examinations in this series, no meaningful comparison between imaging modalities can be made. Decisions regarding additional imaging should therefore remain individualised and based on clinical assessment, multidisciplinary input, and the potential consequences of delaying definitive treatment.

Diagnostic uncertainty and the importance of timely management

A key challenge in suspected appendicitis during pregnancy is balancing the risks of operative intervention against the potential consequences of missed or progressive disease. Concerns regarding fetal wellbeing, diagnostic uncertainty, and the possibility of a negative appendicectomy may influence decision-making. Nevertheless, persistent clinical concern should prompt ongoing reassessment and multidisciplinary review.

The interval from presentation to surgery ranged from one to eight days, with a mean duration of 3.4 days. This finding should be interpreted cautiously, as delays may reflect additional investigations, repeat clinical assessments, initial conservative management, or other patient-specific clinical factors.

All histopathologically confirmed cases experienced at least one complication, including peritonitis (n = 5/9, 55.6%), sepsis (n = 2/9, 22.2%), perforation (n = 1/9, 11.1%), or preterm labour (n = 1/9, 11.1%). These findings are consistent with previously published literature demonstrating poorer outcomes among patients with complicated appendicitis and delayed intervention during pregnancy. Similar concerns regarding maternal morbidity associated with complicated appendicitis have been reported by Ashbrook M et al. [14]. Persistent clinical suspicion therefore warrants reassessment and timely multidisciplinary decision-making, even when imaging findings are negative, inconclusive, or discordant with the overall clinical picture.

Operative management and histopathological findings

All nine patients underwent operative management, including laparoscopic appendicectomy in 6/9 patients (66.7%) and open appendicectomy in 3/9 patients (33.3%). Selection of the surgical approach was influenced by gestational age, clinical presentation, surgeon preference, and intraoperative findings. Previous studies have demonstrated that both laparoscopic and open appendicectomy can be performed safely during pregnancy when appropriately selected [9-15]. However, direct comparison of outcomes remains challenging because published studies differ substantially in patient selection, disease severity, and timing of intervention.

Histopathological examination confirmed acute appendicitis in 5/9 patients (55.6%), resulting in a negative appendicectomy rate of 4/9 patients (44.4%). Despite advances in imaging, this rate remains comparable to rates reported by McGory ML et al. [8] and other published studies evaluating appendicitis in pregnancy [11-15]. These findings highlight the persistent difficulty of achieving diagnostic certainty in pregnant patients presenting with suspected appendicitis. Although avoidance of unnecessary surgery remains desirable, delayed intervention in patients with genuine appendicitis may result in increased morbidity. Consequently, management decisions should be based on the integration of clinical findings, laboratory investigations, imaging results, and ongoing multidisciplinary review.

Clinical implications

The findings of this study support several practical considerations. First, ultrasound should be considered an initial diagnostic investigation rather than a definitive exclusion test when the appendix is not visualised or when clinical suspicion persists. Second, MRI may provide additional diagnostic information following inconclusive ultrasound findings; however, its results should also be interpreted within the broader clinical context. Third, timely senior surgical input and multidisciplinary discussion remain essential when clinical concern persists despite equivocal imaging findings.

The objective is not to prioritise operative management over imaging, nor to disregard imaging findings altogether. Rather, optimal management requires integration of clinical assessment, laboratory parameters, radiological findings, and multidisciplinary decision-making to facilitate timely and appropriate intervention while balancing maternal and fetal considerations.

Limitations

This study has several limitations. The sample size was small and derived from a single centre, limiting the generalisability of the findings. The retrospective design is associated with the inherent limitations of medical record review, including potential documentation bias and incomplete data capture. In addition, the study included only patients who underwent surgical intervention and therefore does not represent all pregnant patients evaluated for suspected appendicitis. The small number of imaging examinations, particularly CT studies, precludes meaningful assessment of diagnostic performance. Larger multicentre studies are required to further evaluate diagnostic pathways, imaging strategies, and maternal-fetal outcomes in this patient population.

## Conclusions

Acute appendicitis during pregnancy remains a diagnostic challenge because clinical symptoms may overlap with physiological changes of pregnancy and imaging findings may be inconclusive. In this retrospective case series of nine patients, delays in diagnosis and surgical intervention were associated with a substantial burden of complications. MRI appeared to provide greater diagnostic utility than ultrasound in selected patients; however, diagnostic uncertainty and a high negative appendicectomy rate persisted despite the use of contemporary imaging modalities.

Pregnancy alone should not result in unnecessary delays in appropriate surgical assessment or intervention when appendicitis is suspected. Timely multidisciplinary assessment involving obstetric, surgical, anaesthetic, and radiological teams remains essential to optimise maternal and fetal outcomes. Larger multicentre studies are required to further evaluate diagnostic pathways and management strategies in this patient population.
